# Equity and determinants in universal health coverage indicators in Iraq, 2000–2030: a national and subnational study

**DOI:** 10.1186/s12939-021-01532-0

**Published:** 2021-08-30

**Authors:** Hiroko Taniguchi, Md Mizanur Rahman, Khin Thet Swe, Md Rashedul Islam, Md Shafiur Rahman, Nadia Parsell, Ashraf Hussain, Kenji Shibuya, Masahiro Hashizume

**Affiliations:** 1 Department of Global Health Policy, School of International Health, Graduate School of Medicine, The University of Tokyo, 7-3-1, Hongo, Bunkyo-ku, Tokyo 113-0033 Japan; 2 Hitotsubashi Institute for Advanced Study (HIAS), Hitotsubashi University, 2-1, Naka, Kunitachi, Tokyo 186-8601 Japan; 3 Research Center for Child Mental Development, Hamamatsu University School of Medicine, 1-20-1, Handayama, Higashi-ku, Hamamatsu, Shizuoka 431-3192 Japan; 4 United Graduate School of Child Development, Osaka University, Kanazawa University, Hamamatsu University School of Medicine, Chiba University and University of Fukui, Osaka, Japan; 5 Department of Family and Community Medicine, College of Medicine, University of Babylon, Babil, Iraq; 6 Soma COVID Vaccination Medical Center, Soma City Hall, 63-3, Kitamachi, Nakamura, Soma, Fukushima, 976-8601 Japan

**Keywords:** Universal health coverage, Equity, Social determinants, Catastrophic health payment, Bayesian analysis

## Abstract

**Background:**

Equity is one of three dimensions of universal health coverage (UHC). However, Iraq has had capital-focused health services and successive conflicts and political turmoil have hampered health services around the country. Iraq has embarked on a new reconstruction process since 2018 and it could be time to aim for equitable healthcare access to realise UHC. We aimed to examine inequality and determinants associated with Iraq’s progress towards UHC targets.

**Methods:**

We assessed the progress toward UHC in the context of equity using six nationally representative population-based household surveys in Iraq in 2000–2018. We included 14 health service indicators and two financial risk protection indicators in our UHC progress assessment. Bayesian hierarchical regression model was used to estimate the trend, projection, and determinant analyses. Slope and relative index of inequality were used to assess wealth-based inequality.

**Results:**

In the national-level health service indicators, inequality indices decreased substantially from 2000 to 2030. However, the wide inequalities are projected to remain in DTP3, measles, full immunisations, and antenatal care in 2030. The pro-rich inequality gap in catastrophic health expenditure increased significantly in all governorates except Sulaimaniya from 2007 to 2012. The higher increases in pro-rich inequality were found in Missan, Karbala, Erbil, and Diala. Mothers’ higher education and more antenatal care visits were possible factors for increased coverage of health service indicators. The higher number of children and elderly population in the households were potential risk factors for an increased risk of catastrophic and impoverishing health payment in Iraq.

**Conclusions:**

To reduce inequality in Iraq, urgent health-system reform is needed, with consideration for vulnerable households having female-heads, less educated mothers, and more children and/or elderly people. Considering varying inequity between and within governorates in Iraq, reconstruction of primary healthcare across the country and cross-sectoral targeted interventions for women should be prioritised.

**Supplementary Information:**

The online version contains supplementary material available at 10.1186/s12939-021-01532-0.

## Introduction

Achieving universal health coverage (UHC) is a global health priority and one of the major targets of the Sustainable Development Goals (SDGs) [1]. UHC enables all citizens to have access to quality health services without financial risk when they use those services [1]. Under SDG3, World Health Organisation (WHO) and The World Bank defined a set of UHC targets for the United Nations (UN) member states to achieve by 2030: (1) at least 80% essential health service coverage for the entire population of the country irrespective of economic status, gender, and place of residence (equity); and (2) 100% protection from catastrophic and impoverishing health payments by 2030 [2, 3]. Both service coverage and financial risk protection indicators should be measured with a focus on equity (population coverage).

The latest UHC global monitoring report found all regions and all income groups to have made progress toward UHC, with the greatest progress seen in lower income countries due to lower baseline coverage and success in communicable disease intervention and maternal and child health [3]. However, equity continues to be a major challenge. For example, globally, there is an about 40% gap in coverage for at least four antenatal care visits (ANC4) between poorest and richest households [3]. Moreover, national averages of coverage indicators are likely to mask significant within-country variations of coverage indicators by socioeconomic and sociodemographic strata which have been generated in the context of each county. Therefore, it is important to investigate those variations and their magnitudes in all three dimensions of UHC.

Since the 1970s, Iraq has suffered from a series of wars, conflicts, and political turmoil which damaged the healthcare systems and other essential infrastructure [4]. Iraq’s Ministry of Health developed the National Health Policy 2014–2023, where UHC was its core element, even before the adoption of the SDGs. However, the healthcare systems have not been fully restored and free public health services are not equitably distributed across governorates [5]. The country has not developed a pre-pooled financing mechanism and the share of out-of-pocket (OOP) health spending in total health expenditure increased from 29% in 2004 to 78% in 2016 [6]. In addition, the violence and insecurity created a number of internally displaced persons (IDPs) and refugees (as of 31 August 2020, 1.4 million IDPs in and out of camps and 4.7 million returnees) [7]. In response to the mounting humanitarian needs and to secure the progress towards UHC, Health Cluster, an inter-organisational health partnership in humanitarian emergencies, has worked on Health Emergencies Programme and Essential Package of Health Services (EPHS) expansion with Iraq MoH and other actors [8]. However, worsened trends in health service coverage and financial risk protection were identified in both conflict-affected and underdeveloped governorates [9]. As Iraq has embarked on a new reconstruction process since 2018, it is important to understand the trend and progress toward UHC with a focus on equity and to aim for equitable healthcare access across the country.

To date, many studies have assessed injuries and/or deaths in emergencies in Iraq and some studies have assessed a limited number of UHC-related indicators in Iraq using specific data points [3, 10–15]. However, no study has comprehensively assessed progress toward UHC in Iraq by focusing on equity strata, at the national and subnational levels, by place of residence, education, wealth, and other sociodemographic characteristics, using nationally representative survey data. This study is the first to examine the inequality and determinants associated with Iraq’s progress towards UHC targets in 2030.

## Methods

### Data sources

To estimate trends and projections of UHC indicators including inequality and determinants, we used data from six nationally representative population-based household surveys in Iraq. For the estimation of coverage of health service indicators, we employed Multiple Indicator Cluster Survey (MICS) in 2000, 2006, 2011, and 2018; and for the assessment of financial risk protection indicators, we used Household Socio-Economic Survey (HSES) in 2006–2007 and 2012. All included surveys employed a multi-stage cluster sampling design and had high response rates (more than 97%). A brief description of these surveys is presented in Appendix (table A1).

### Measurement of UHC

Following the WHO guidelines and reviewing data availability, we included 14 indicators including maternal- and child-health and environmental indicators in the study (Appendix table A2). In accordance with previous studies, financial hardship was assessed with two indicators: incidence of catastrophic health expenditure and incidence of impoverishment [16]. A household’s OOP payments for healthcare are regarded as catastrophic health expenditure if it exceeds a certain threshold value of either total household consumption, non-food consumption or a household’s capacity to pay [16]. For our study, we used 10% of total household consumption expenditure to estimate incidence of catastrophic health expenditure at the national-, subnational- and place-of-residence-levels. Health expenditure is considered as impoverishing when a non-poor household becomes poor due to OOP payments for healthcare [16]. The details of the measurement procedure for catastrophic health expenditure and impoverishment are in Appendix (Sect. 3).

### Predictor variables

Considering the contexts of Iraq, this study aimed to reflect the associations between displacement and health in assessing UHC trends and projections in Iraq. After examining possible variables and their data availability, for health service trend and projection analysis, we selected three predictor variables: the numbers of IDP (at the subnational level), population density (at the subnational level), and total health expenditure per capita (at the national level) from 2000 to 2018. The steps to identify feasible predictor variables for health service trend and projection analysis, the detailed data source of IDP, and the definition of population density are described in Appendix (Sect. 4). For determinant analysis, we used a range of predictor variables based on the health service and financial risk protection indicators which were behind the targets. Following previous guidelines and studies, we include place of residence, household wealth quintile, gender of household head, age and education of women and household head, number of ANC visits, gender and birth order of the last newborn, and number of household members aged under 5 and over 65 years old [17–19]. These predictor variables and their definitions are presented in Appendix (table A5.1 and A5.2).

### Statistical analysis

We developed Bayesian hierarchical regression models to project predictor variables and to estimate the trend in, and projection of, health service indicators up to 2030 by governorate, residence, and wealth, separately. Bayesian approach addresses the issues of limited data points across all provenances and is used and favoured when we aim to project probabilities. Ecological modelling is characterised by high uncertainty because of complex and often unknown cause-effect relationships among variables. Therefore, a probabilistic approach is needed to yield distributions of possible outcomes. Bayesian method also has an ability to combine prior knowledge about parameters with evidence from data and is favoured for analysis of hierarchical models [20]. These advantages of Bayesian approach were key to conducting our study. The details including the descriptions of the models are presented in Appendix (Sect. 6). The governorate-level mean, residence-level mean, and quintile-level mean were assumed to be normally distributed and non-informative prior was applied. The model assumed that the effects of predictors were the same across governorates. The predictor variables were determined based on the previous literature, correlation, and Deviance Information Criteria (DIC). The details of the assessment of convergence of Markov chain Monte Carlo (MCMC) output for each of the Bayesian models and examination of the validity of the models were also described in Appendix (Sect. 6). For the determinant analysis, we used individual- and household-level original dataset. We estimate adjusted odds ratios (ORs) and 95% Credible Interval (CrI) for each health service and financial risk protection indicator using a Bayesian hierarchical regression model. For health service indicators, this study used determinant analysis to identify the factors which may be related to the low coverage of health service indicators in the recent year (2018) as this may have an impact on the trends in the future. We selected health service indicators which did not reach the 80% target by 2030 in the prior study: family planning needs satisfied, ANC4, full immunisation, ARI treatment, and oral rehydration therapy [9].

Wealth-based inequalities in health service coverage and incidence of catastrophic health expenditure were performed using slope index of inequality (SII) and relative index of inequality (RII). SII measures the absolute difference in intervention coverage or catastrophic health expenditure between the richest households and the poorest households. A positive SII value indicates that rich households have either higher intervention coverage or higher financial catastrophe than poor households. RII is a weighted measure of inequality which represents the ratio of estimated values of a health indicator of the richest households to the poorest households. An RII value greater than 1 indicates pro-rich inequality and a value smaller than 1 indicates pro-poor inequality. The detailed methods of SII and RII are described in Appendix (Sect. 6).

Stata/SE version 15 was used for data management. Bayesian models were developed in JAGS version 4.3.0 and implemented in R version 3.6.1.

## Results

### Inequalities in health service indicators

**Fig. 1:**
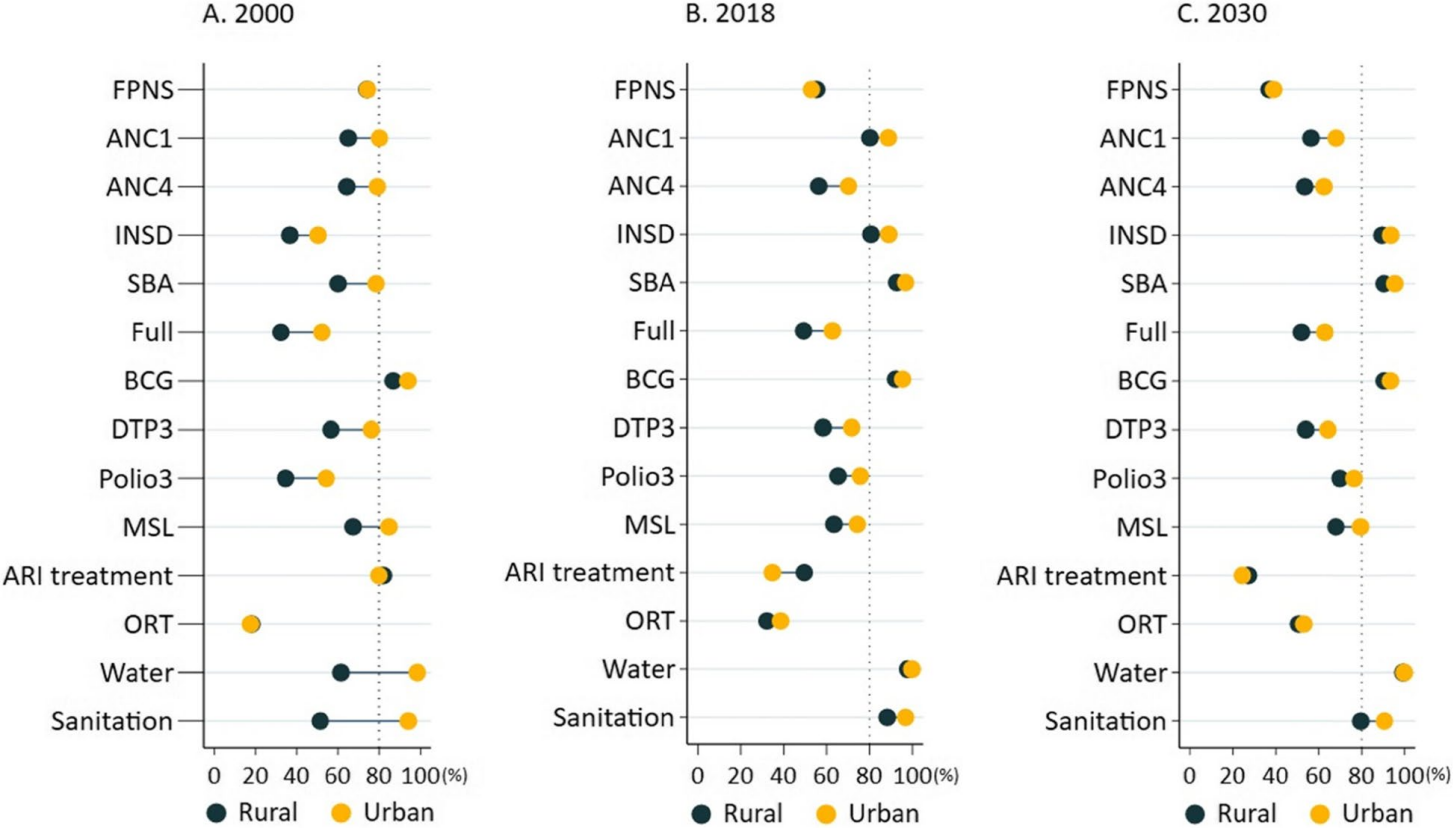
Residence-specific health service indicators at the national level in Iraq, 2000–2030. *FPNS* family planning needs satisfied, *ANC1* at least one antenatal care visit, *ANC4* at least four antenatal care visits, *INSD* institutional delivery, *SBA* skilled birth attendance, *Full* full immunisation, *MSL* measles immunisation, *ARI treatment* acute respiratory infection treatment for pneumonia, *ORT* oral rehydration therapy, *Water* improved water sources, *Sanitation* adequate sanitation

Figure 1 shows the residence-specific coverage of health service indicators at the national level in Iraq from 2000 to 2030. Of the 14 indicators, only institutional delivery, skilled birth attendance (SBA), BCG, improved water sources, and adequate sanitation at both urban and rural areas are projected to achieve the 80% coverage in 2030. Measles will reach the target by 2030 only in urban area. The coverage of all health service indicators in rural areas was lower than that in urban areas. Although those coverage disparities between rural and urban areas decreased over time, ANC1, DTP3, measles, full immunisation and adequate sanitation will maintain at least a 10 percentage-point gap in 2030.

**Fig. 2:**
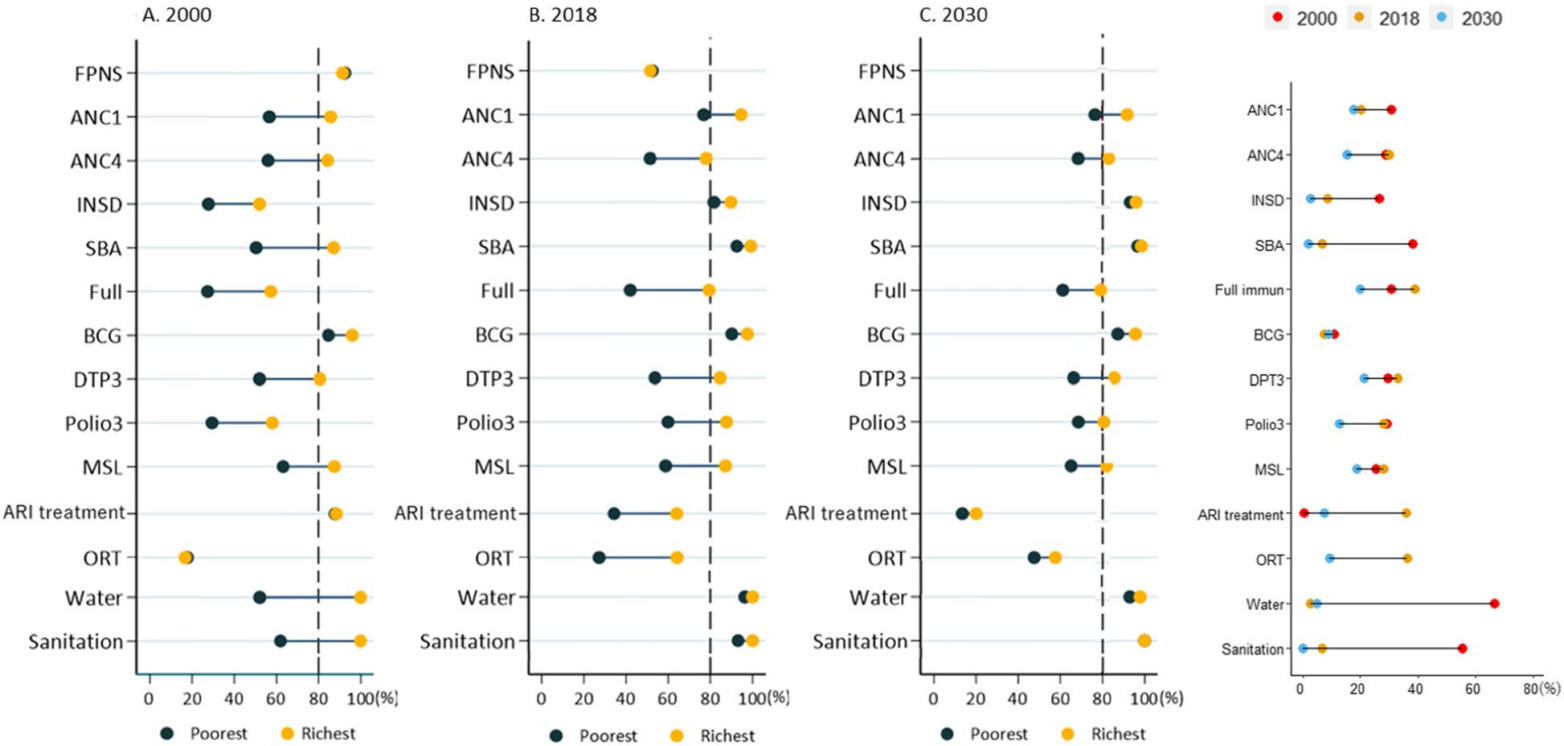
Wealth quintile-specific health service indicators at the national level; Slope index of inequality in the national-level health service indicators (% point) in Iraq, 2000–2030. *FPNS* family planning needs satisfied, *ANC1* at least one antenatal care visit, *ANC4* at least four antenatal care visits, *INSD* institutional delivery, *SBA* skilled birth attendance, *Full (immun)* full immunisation, *MSL* measles immunisation, *ARI treatment* acute respiratory infection treatment for pneumonia, *ORT* oral rehydration therapy, *Water* improved water sources, *Sanitation* adequate sanitation

Figure 2 shows national-level health service indicators by wealth quintile and the corresponding SII from 2000 to 2030. Overall, the inequality gap between the poorest and richest quintiles decreased over thirty years for all health indicators. However, the coverage of most health indicators in the poorest quintile was substantially lower than the richest group in all years. By 2030, institutional delivery, SBA, BCG, improved water sources, and adequate sanitation are projected to achieve the 80% target in both quintiles. All the health service indicators in the richest group except childhood disease treatments are predicted to achieve the 80% target by 2030. As shown in the rightmost panel of Fig. 2, inequality indices decreased substantially from 2000 to 2030. However, several indicators will continue to have a wide gap in 2030. The widest inequalities are found in DTP3 (21.4 percentage points) in 2030, followed by full immunisation (20.0 percentage points), measles (18.6 percentage points), ANC1 (17.6 percentage points), and ANC4 (15.4 percentage points). The lowest inequality was found in adequate sanitation (0.0 percentage points), SBA (1.8 percentage points) and institutional delivery (2.8 percentage points). The value for SII for health service coverage from 2000 to 2030 is presented in Appendix (Table A7).

In the determinant analysis, we selected the following health service indicators: family planning needs satisfied, ANC4, full immunisation, ARI treatment, and oral rehydration therapy. As shown in the Table 1, maternal education level and household wealth quintile increased ORs of ANC4 and full immunisation coverage; an inverse association was found for birth order. The number of ANC visits were positively associated with ARI treatment. The ORs of ANC4, full immunisation, and oral rehydration therapy were lower for rural residents than for urban residents.

**Table 1:** Determinants of family planning, antenatal care, full immunisation and disease treatment coverage in Iraq, 2018

Characteristics	Odds Ratio (95% Credible Interval)				
	**FPNS**	**ANC4**	**Full immunisation**	**ARI treatment**	**Oral rehydration therapy**
Mother age, years					
< 20	1.00	1.00	1.00	1.00	1.00
20–35	1.12 (1.03–1.22)	1.04 (0.78–1.33)	0.49 (0.36–0.63)	0.86 (0.48–1.44)	0.28 (0.19–0.40)
≥ 36	1.31 (1.21–1.42)	1.09 (0.80–1.46)	0.79 (0.67–0.93)	0.93 (0.66–1.26)	0.53 (0.39–0.70)
Mother’s education					
No education	1.00	1.00	1.00	1.00	1.00
Primary	0.99 (0.88–1.11)	1.40 (1.21–1.63)	1.43 (1.19–1.70)	0.92 (0.62–1.33)	1.27 (0.98–1.62)
Secondary	0.97 (0.84–1.13)	1.62 (1.34–1.95)	1.60 (1.33–1.93)	1.29 (0.77–2.10)	1.21 (0.83–1.72)
Higher	0.92 (0.80–1.06)	1.95 (1.65–2.27)	1.76 (1.39–2.20)	1.50 (0.79–2.66)	1.59 (1.19–2.09)
Antenatal care visits					
0	NA	NA	1.00	1.00	1.00
1	NA	NA	0.96 (0.77–1.19)	5.33 (1.89–11.86)	0.49 (0.26–0.85)
2	NA	NA	1.01 (0.82–1.21)	7.34 (2.82–15.31)	0.73 (0.50–1.02)
3	NA	NA	0.97 (0.78–1.20)	37.23 (9.68–95.20)	1.01 (0.71–1.38)
4	NA	NA	1.54 (1.24–1.91)	11.34 (4.50–23.07)	NA
Birth order					
0	1.00	NA	1.00	1.00	1.00
1	0.41 (0.36–0.46)	1.00	0.92 (0.81–1.04)	0.72 (0.44–1.04)	0.96 (0.70–1.28)
2–3	0.78 (0.71–0.86)	0.52 (0.43–0.60)	0.57 (0.47–0.68)	1.22 (0.71–2.01)	1.37 (0.95–1.88)
> = 4	NA	0.37 (0.30–0.45)	0.61 (0.55–0.69)	1.15 (0.73–1.72)	1.14 (0.87–1.46)
Gender of the last newborn					
Male	NA	NA	1.00	1.00	1.00
Female	NA	NA	0.82 (0.69–0.97)	1.01 (0.68–1.44)	1.16 (0.89–1.48)
Household wealth quintile					
Q1 (Poorest)	1.00	1.00	1.00	1.00	1.00
Q2	1.00 (0.88–1.14)	1.47 (1.29–1.66)	1.22 (0.98–1.52)	0.37 (0.24–0.54)	0.74 (0.53–1.01)
Q3	0.85 (0.75–0.97)	1.37 (1.27–1.48)	1.12 (0.91–1.37)	0.58 (0.37–0.88)	0.78 (0.55–1.10)
Q4	0.90 (0.78–1.03)	1.56 (1.34–1.81)	1.53 (1.21–1.92)	0.60 (0.31–1.07)	0.70 (0.45–1.04)
Q5 (Richest)	0.75 (0.62–0.90)	2.41 (1.96–2.92)	2.43 (1.87–3.18)	1.03 (0.62–1.77)	0.90 (0.63–1.25)
Place of residence					
Urban	1.00	1.00	1.00	1.00	1.00
Rural	1.02 (0.91–1.14)	0.72 (0.62–0.82)	0.79 (0.64–0.96)	1.35 (0.94–1.87)	0.69 (0.52–0.90)
Variance (cov.)					
Governorate	0.30 (0.13–0.64)	0.17 (0.07–0.34)	0.68 (0.03–1.41)	0.48 (0.47–1.44)	0.48 (0.13–1.18)
Cluster	0.11 (0.04–1.19)	0.34 (0.21–0.49)	0.64 (0.10–1.02)	1.79 (0.18–4.32)	1.02 (0.40–1.90)

*Note*: *NA* Not applicable, *FPNS* Family planning needs satisfied, *ANC4* At least four antenatal care visits, *ARI treatment* acute respiratory infection treatment for pneumonia

### Inequalities in financial risk protection indicators

Figure A8 in Appendix shows the residence-specific incidence of catastrophic health expenditure at the national and subnational (governorate) level in 2007 and 2012. The urban–rural gap in catastrophic health expenditure for most governorates, except Najaf, Thiqar and Babil, was quite small in 2007. However, the gap increased substantially in 2012 in some selected governorates, especially in Duhok, Erbil, Wasit, Salahaddin and Muthana. In 2012, two different trends in the incidence of catastrophic health expenditure were observed: in Duhok, Erbil, Wasit, Muthana, and Basrah, rural residents experienced more incidents than urban residents; in Salahaddin, Missan, Karbala, Najaf and Nainawa, urban residents incurred more catastrophic health expenditure than rural residents.

The results of SII and RII for catastrophic health expenditure at the national and governorate levels and the observed proportion of catastrophic health expenditure for each quintile by governorate are presented in Table 2 and Appendix (figure A9.1 and A9.2, respectively). Overall, pro-rich absolute inequality in catastrophic health expenditure increased more than fourfold at national, urban and rural areas. From 2007 to 2012, the SII value increased from 3.1 percentage points to 13.7 in urban areas and increased from 2.2 to 9.1 in rural areas, respectively.

**Table 2:** Slope and relative index of inequality for catastrophic health expenditure by residence and governorate in Iraq, 2007 and 2012

Country / Residence / Governorate	2007		2012	
	**SII (95% CI)**	**RII (95% CI)**	**SII (95% CI)**	**RII (95% CI)**
National	2.3 (1.3–3.2)	1.7 (1.3–2.1)	11.5 (10.0–12.9)	2.4 (2.1–2.6)
Place of residence				
Urban	3.1 (1.9–4.2)	2.2 (1.6–2.8)	13.7 (11.8–15.6)	2.8 (2.4–3.2)
Rural	2.2 (0.4–3.9)	1.6 (1.0–2.2)	9.1 (6.8–11.4)	2.0 (1.6–2.3)
Governorate				
Anbar	1.9 (-1.4–5.1)	2.1 (-0.5–4.8)	11.6 (5.8–17.4)	2.4 (1.3–3.5)
Babil	2.3 (-1.5–6.0)	1.9 (-0.1–4.0)	4.4 (-3.6–12.3)	1.3 (0.6–2.0)
Baghdad	6.5 (2.7–10.3)	4.8 (0.8–8.8)	17.0 (12.2–21.9)	4.9 (2.7–7.0)
Basrah	3.7 (-0.9–8.3)	1.9 (0.4–3.3)	6.2 (0.5–12.0)	1.6 (0.9–2.3)
Diala	1.7 (-1.1–4.5)	2.2 (-0.4–4.8)	16.6 (10.1–23.1)	3.2 (1.8–4.7)
Duhok	-2.6 (-7.5–2.4)	0.6 (0.0–1.2)	6.5 (-1.0–14.0)	1.4 (0.9–1.9)
Erbil	1.2 (-3.3–5.8)	1.3 (0.1–2.5)	16.8 (10.6–23.1)	2.3 (1.6–3.0)
Karbala	0.4 (-2.3–3.1)	1.2 (-0.1–2.4)	15.7 (6.5–24.8)	5.5 (0.2–10.7)
Kirkuk	5.2 (1.1–9.3)	4.4 (-0.1–8.8)	6.3 (-2.7–15.2)	1.4 (0.7–2.1)
Missan	1.3 (-3.1–5.7)	1.4 (0.0–2.7)	21.2 (14.8–27.6)	8.0 (3.2–12.3)
Muthana	1.7 (-2.3–5.7)	1.6 (-0.1–3.2)	4.9 (-2.2–12.0)	1.6 (0.5–2.7)
Nainawa	2.0 (-2.2–6.1)	1.5 (0.2–2.8)	6.9 (2.3–11.4)	2.1 (1.1–3.1)
Najaf	0.1 (-4.7–4.9)	1.0 (0.1–1.9)	7.7 (-0.6–16.0)	2.0 (0.6–3.3)
Qadissiyah	3.3 (-2.9–9.5)	1.5 (0.4–2.5)	6.4 (-1.6–14.3)	1.7 (0.6–2.7)
Salahaddin	0.4 (-1.9–2.7)	1.2 (0.0–2.4)	13.3 (7.9–18.7)	3.2 (1.7–4.7)
Sulaimaniya	1.0 (-3.3–5.2)	1.3 (-0.1–2.6)	-0.2 (-4.3–3.8)	1.0 (0.7–1.3)
Thiqar	1.6 (-2.6–5.8)	1.4 (0.1–2.8)	11.5 (5.0–18.0)	3.2 (1.2–5.2)
Wasit	4.7 (0.9–8.6)	4.2 (-0.2–8.5)	8.1 (1.1–15.1)	1.7 (0.9–2.4)

*SII* Slope index of inequality, *RII* Relative index of inequality, *CI* Confidence interval

In most of the governorates, the incidence of catastrophic health expenditure in 2007 and 2012 was higher in the richest quintile than the poorest quintile, except for Duhok where pro-poor inequality in 2007 shifted to pro-rich in 2012 and Sulaimaniya where pro-rich inequality in 2007 shifted to pro-poor in 2012. The pro-rich inequality gap in catastrophic health expenditure increased substantially in all governorates except Sulaimaniya from 2007 to 2012. The highest increase in pro-rich inequality from 2007 to 2012 was in Missan (1.3 to 21.2 percentage points), followed by Karbala (0.4 to 15.7 percentage points), and Erbil and Diala (around 2 to 17 percentage points). In 2012, the lowest inequality in catastrophic health expenditure observed was Sulaimaniya (-0.2 percentage points), followed by Babil (4.4 percentage points) and Muthana (4.9 percentage points).

Table 3 presents the estimated ORs and 95% credible interval (Crl) for ORs for catastrophic health expenditure and impoverishment. Determinant analysis of financial risk protection indicated that the higher number of elderly people per household increased OR of incurring catastrophic health expenditure (1.44 [95% CrI 1.36–1.53]) and that the higher number of children under 5 years had increased OR of impoverishment (1.20 [95% CrI 1.15–1.25]). Households in the richest quintile were more than twice likely to incur catastrophic health expenditure compared to the poorest. Higher education level of household heads decreased ORs of catastrophic health expenditure and impoverishment. Households in rural areas had increased ORs of catastrophic health expenditure and impoverishment compared to their urban counterparts.

**Table 3:** Determinants of financial risk protection in Iraq, 2007 and 2012

Characteristics	Odds Ratio (95% Credible Interval)	
	**Catastrophic health expenditure**	**Impoverishment**
Household head age	1.00 (1.00–1.00)	1.00 (0.99–1.00)
Household head gender		
Male	1.0	1.0
Female	1.21 (1.09–1.34)	0.87 (0.68–1.10)
Household head’s education ^a^		
No education	1.00	1.00
Primary	1.04 (0.95–1.13)	0.77 (0.64–0.92)
Secondary	0.71 (0.61–0.81)	0.40 (0.31–0.52)
Higher	0.68 (0.57–0.81)	0.21 (0.17–0.26)
Other	0.92 (0.81–1.04)	3.68 (1.62–6.73)
Household member		
Number of under-5	0.99 (0.96–1.02)	1.20 (1.15–1.25)
Number of over-65	1.44 (1.36–1.53)	1.09 (0.93–1.26)
Household wealth quintile		
Q1 (Poorest)	1.00	
Q2	1.31 (1.25–1.38)	NA
Q3	1.51 (1.42–1.61)	NA
Q4	1.79 (1.67–1.91)	NA
Q5 (Richest)	2.46 (2.30–2.62)	NA
Place of residence		
Urban	1.00	1.00
Rural	1.17 (1.10–1.25)	1.22 (1.06–1.39)
Survey year		
2007	1.00	1.00
2012	4.17 (3.86–4.49)	1.54 (1.33–1.80)
Variance (cov.)		
Level 2 (cluster)	0.32 (0.23–0.39)	0.16 (0.04–0.42)
Level 3 (governorate)	0.06 (0.03–0.13)	0.06 (0.02–0.13)

*Note*: *NA* Not applicable; ^a^ No education means Do not read and write, read only, can read and write or no diploma. Primary includes elementary and intermediate (mid school). Secondary includes preparatory, vocational, and institute diploma. Higher includes bachelor’s degree, high diploma, master’s degree, or doctorate degree

## Discussion

This study highlights the progress toward UHC in Iraq with a focus on equity strata, at the national and subnational levels, by place of residence, education, wealth, and other sociodemographic characteristics. Inequality in health service indicators by place of residence and wealth quintile decreased from 2000 to 2030, however, the magnitude of inequality will remain large in ANC visits and childhood immunisations in 2030. Pro-rich inequality in catastrophic health expenditure increased more than fourfold in urban and rural areas from 2007 to 2012. Education level, household wealth, and place of residence were common key determinants of health service and financial risk protection indicators. Mothers’ higher education and more ANC visits were possible factors for increased coverage of health service indicators.

The inequality analyses of health service coverage in 2018 present the current serious pro-urban and pro-rich inequalities in ANC visits, child immunisations, and childhood disease treatment in Iraq. These results are also in line with our findings from the determinant analyses and other studies [21–24]. Moving towards 2030, the overall inequalities by place of residence and by wealth in Iraq will continue to narrow mainly because of improvements in rural areas and among poorer quintiles, as seen in multiple countries such as Pakistan, Sierra Leone, and Malawi [23]. Reductions in inequalities may be a result of Essential Package of Health Services (EPHS) for Iraq and other external assistance prioritising vulnerable populations as humanitarian targets in efforts to strengthen access to essential health services. Nonetheless, pro-urban and pro-rich trends are projected to remain for ANC visits and child immunisations except BCG. Rural residents and those among the poorest quintile will achieve the 80% target in only five health service indicators in 2030.

Mother’s education, number of ANC visits, and birth order of the last-born child were significant factors for the coverage of several health service indicators. Consistent with other studies [21, 22, 25, 26], this study found that higher education in mothers would lead to better health service coverage for themselves (ANC visits) and their children (immunisations and disease treatment). The fluctuating literacy rates in Iraq indicate fragile opportunities for education. Moreover, interventions such as educational campaigns for maternal and child health have been affected by escalated violence and insecurity. Improvements in mother’s education provide not only the ability to understand and gain necessary knowledge but also open up income-earning opportunities for women. Therefore, cross-sectoral efforts including health-education and health-finance could increase women’s access to healthcare services [27–29]. This study found that more ANC visits were associated with better coverage of ARI treatment for children. This may indicate that the more opportunities to acquire healthcare knowledge mothers have, the more proactively they seek care for their children. Instabilities remain in the post-conflict Iraq. On top of cross-sectoral recovery efforts for essential infrastructure, focused campaigns and participatory interventions for women, particularly female heads of the households, could be considered for increasing awareness and interests in proactive care-seeking behaviour [24].

Both urban and rural residents experienced a fourfold increase in catastrophic health expenditure from 3% in 2007 to around 12% in 2012. Determinant analysis indicates that wealthier households, rural residence, households with more children under 5 years and/or elderlies, and female household head had an increased risk of financial hardship. Similarly, pro-rich inequality in catastrophic health expenditure was found in other middle-income counties such as India, while pro-poor inequality was observed in Bangladesh [19, 30, 31]. Common reasons for the increased financial risks and pro-rich inequalities could be due to unequal pro-rich distribution of the wealth and over-dependency of OOP payments for healthcare service (78% in 2016), alongside low GDP spending on health and absence of risk pooling mechanism in health financing system [5, 6, 18, 30]. Moreover, young children and elderly people are vulnerable to diseases and injuries, which can be exacerbated by long-lasting brutal lifestyle brought on by insecurity, deprivation and/or displacement.

Although our findings present urban and rural residents were exposed to a similar level of financial risks at the national level, we found wide inequalities in catastrophic health expenditure between urban–rural in most of the governorates in 2012. Substantial incidence gaps between urban–rural were found in Duhok, Erbil, and Wasit, where rural residents incurred more financial catastrophe than their urban counterparts. However, urban residents in Salahaddin, Najaf, Nainawa, and Missan incurred more catastrophic health expenditure than rural residents. From 2007 to 2013, Iraq went through reconstruction, where the benefits from reconstruction investment may have been distributed unequally. For example, in Duhok, a most developed governorate, rural residents may have been left behind in economic development [32]. On the other hand, Missan, one of the most underdeveloped governorates, was left out of the economic boost and the urban residents might face greater catastrophic health expenditure than the rural residents who originally had limited health service options [33].

Regarding wealth-based inequity in catastrophic health expenditure among the governorates, pro-rich inequality dominated the governorates heavily affected by the Iraq War-related violence and the most underdeveloped governorates suffering from poverty [33]. The war-affected governorates such as Baghdad, Salahaddin, Anbar, Erbil, and Diala received reconstruction support; however, it may not have been distributed equitably and may have brought economic inequality even within a governorate [34]. Our findings indicate that the richer quintiles faced more catastrophic health expenditure most likely due to having more means to pay for health services. Nonetheless, poorer quintiles should be paid attention to, as they may forgo needed healthcare due to inability to pay. Furthermore, Iraq has had an overconcentration of health services in the capital and health workers involved in dual practice [5]. Conflict-caused destructed infrastructure, displacement, and brain-drain could have magnified shortages in health resources, inequitable distribution of healthcare, and weak financial protection.

Despite limited studies on the effectiveness of interventions for improving equity in maternal or child health, several additional points are argued to promote reduction of inequity [35]. ANC visits and child immunisations require multiple service contacts; therefore, especially, knowledge/information-sharing, service provision by community health workers and/or by outreach, and financial support could be considered [21, 27, 35–38]. Financial support may be achieved through an incentive scheme like a conditional cash transfer scheme and voucher scheme as well as equalising health insurance scheme as a long-term measure [35–38]. Particularly in unstable settings and/or displacement, sustainable and timely care-seeking is a challenge. Special attention to rural residents or isolated populations, including helpless returnees and indigents, should be maintained in health strategies and external aid interventions [24, 39, 40]. It is also critical to distribute reconstructed primary healthcare facilities and human resources widely, with aims to dissolve serious inequity and achieve UHC [41].

This study has several strengths. First, this is the first study which assessed progress toward UHC in Iraq by focusing on equity strata. Second, this study utilised a large number of population-based, household health and expenditure survey data. Third, this study applied population movement of IDP and population density in each governorate to the estimates. Finally, this study used Bayesian hierarchical regression models in the trend, projection, and determinant analysis. However, this study has some limitations. All of the results were based on present trends and estimations where adjusted or additional policies and interventions will not be implemented. In the determinant analysis, our models may have missed unobservable factors, such as occupation, ethnicity, and socio-political and structural determinants. Due to scarce data, this study was not able to assess management of other communicable and non-communicable diseases, service capacity and access, and projection of financial risk protection indicators.

## Conclusions

Inequity in health service indicators by place of residence and wealth quintile are projected to decrease from 2000 to 2030 in Iraq. However, the magnitude of inequity will remain large in ANC visits and childhood immunisations in 2030. Education level, household wealth, and place of residence are common key determinants of health service and financial risk protection indicators. Urgent health-system reform including enhanced antenatal care and child immunisations and nation-wide measures to improve financial risk protection should be prioritised to reduce inequity, with consideration for vulnerable households having female-heads, less educated mothers, and more children and/or elderly people. Considering varying inequity between and within governorates in Iraq, reconstruction of primary healthcare across the country and cross-sectoral targeted interventions for women should be critical.

## Supplementary Information

**Additional file1: Appendix section 1**. Survey characteristics. **Appendix section 2**. Health service indicators. **Appendix section 3**. The definitions of household consumption expenditure and out-of-pocket health payment, estimation of financial burden and incidence of catastrophic health expenditure at different threshold. **Appendix section 4**: Predictor variables for trend and projection analysis and data of internally displaced people and definition of population density. **Appendix section 5**. Predictor variables used in the determinant analyses. **Appendix section 6**. Statistical analysis. **Appendix section 7**. National-level health service coverage: slope index of inequality. **Appendix section 8**. Subnational-level catastrophic health expenditure by place of residence. **Appendix section 9**. Subnational-level catastrophic health expenditure: slope index of inequality and wealth quintile-specific incidence.

## Data Availability

All individual-level data are available from UNICEF Multiple Indicator Cluster Survey (MICS) website (https://mics.unicef.org/surveys). Household data are available from The World Bank Household Socio-Economic Survey (HSES) website (https://microdata.worldbank.org/index.php/catalog/lsms). Therefore, interested researcher could access all data from the websites. All data generated or analysed during this study are included in this published article and its supplementary information files. The study protocol is available from the corresponding author.
