# Supplementary material for: Equity and determinants in universal health coverage indicators in Iraq, 2000–2030: a national and subnational study
Source: Int J Equity Health. 2021 Aug 30;20:196. doi: 10.1186/s12939-021-01532-0 (PMC8404248; doi:10.1186/s12939-021-01532-0)
Supplement: Supplementary file 1 — Additional file1: Appendix section 1. Survey characteristics. Appendix section 2. Health service indicators. Appendix section 3. The definitions of household consumption expenditure and out-of-pocket health payment, estimation of financial burden and incidence of catastrophic health expenditure at different threshold. Appendix section 4: Predictor variables for trend and projection analysis and data of internally displaced people and definition of population density. Appendix section 5. Predictor variables used in the determinant analyses. Appendix section 6. Statistical analysis. Appendix section 7. National-level health service coverage: slope index of inequality. Appendix section 8. Subnational-level catastrophic health expenditure by place of residence. Appendix section 9. Subnational-level catastrophic health expenditure: slope index of inequality and wealth quintile-specific incidence. [file 12939_2021_1532_MOESM1_ESM.docx]

**Supplementary appendix**

**Equity and determinants in universal health coverage indicators in Iraq, 2000-2030: a national and subnational study**

Appendix section 1. Survey characteristics

Appendix section 2. Health service indicators

Appendix section 3. The definitions of household consumption expenditure and out-of-pocket health payment, estimation of financial burden and incidence of catastrophic health expenditure at different threshold

Appendix section 4: Predictor variables for trend and projection analysis and data of internally displaced people and definition of population density

Appendix section 5. Predictor variables used in the determinant analyses

Appendix section 6. Statistical analysis

Appendix section 7. National-level health service coverage: slope index of inequality

Appendix section 8. Subnational-level catastrophic health expenditure by place of residence

Appendix section 9. Subnational-level catastrophic health expenditure: slope index of inequality and wealth quintile-specific incidence

References

**Appendix section 1. Survey characteristics**

**Table A1: Survey characteristics ^a^**

| **Survey** | **Year** | **Sample design** | **Number of households** | **Response rate** |
| --- | --- | --- | --- | --- |
| MICS | 2000 ^b^ | Three-stage, stratified random sampling | 13,011 | 99.2% |
|  | 2006 ^b^ | Multi-stage, stratified cluster sampling | 17,873 | 99.4% |
|  | 2011 ^b^ | Multi-stage, stratified cluster sampling | 35,701 | 99.6% |
|  | 2018 ^b^ | Multi-stage, stratified cluster sampling | 20,214 | 99.5% |
| HSES | 2007 | Two-stage, stratified cluster sampling | 17,513 | 97.6% |
|  | 2012 | Two-stage stratified cluster sampling | 24,944 | 97.9% |

Note: ^a^ All information taken from survey reports. ^b^ For the purposes of MICS, internally displaced persons living in United Nations/government notified camps, military installations, and non-residential units such as business establishments were not considered in the scope of the survey [1].

MICS, Multiple Indicator Cluster Survey; HSES, Household Socio-Economic Survey

**Appendix section 2. Health service indicators**

**Table A2: Health service indicators**

| **Indicators** |  | **Definition** |
| --- | --- | --- |
| **Environment (2)** | | |
| Improved water sources |  | The proportion of households whose main source of drinking water is an improved source, including piped water, tube well/tube-hole, protected well, protected spring, rain water collection, tanker truck, cart with small tank, water kiosk, bottled water and desalinised & sterilised water |
| Adequate sanitation |  | The proportion of households with improved toilet facilities, including flush toilets with piped to sewer system, septic tank, or Don't know where as well as pit latrines with slab, ventilated improved pit latrine, or composting toilet |
| **Maternal health (5)** | | |
| Family planning needs satisfied |  | The proportion of married women aged 15-49 who do not want any more children or want to wait 2 or more years before having another child and are using modern contraception |
| ANC1 |  | The proportion of women age 15-49 years with a live birth in the last 2 years who during the pregnancy of the most recent live birth were attended at least once by skilled health personnel |
| ANC4 |  | The proportion of women age 15-49 years with a live birth in the last 2 years who during the pregnancy of the most recent live birth were attended at least four times by skilled health personnel |
| Institutional delivery |  | The proportion of women age 15-49 years with a live birth in the last 2 years whose most recent live birth was delivered in a health facility |
| Skilled birth attendance |  | The proportion of women age 15-49 years with a live birth in the last 2 years whose most recent live birth was attended by skilled health personnel (doctor, nurse or midwife) |
| **Child heath (7)** | | |
| BCG |  | The proportion of children age 12-23 months who received BCG containing vaccine at any time before the survey |
| DTP3 |  | The proportion of children age 12-23 months who received three doses of DPT vaccine (‎diphtheria, pertussis, tetanus) before the survey |
| Polio3 |  | The proportion of children age 12-23 months who received three doses of polio vaccine before the survey, regardless of whether IPV or OPV |
| Measles |  | The proportion of children age 12-23 months vaccinated against measles at any time before the survey |
| Full immunisation |  | The proportion of children age 12-23 months who received three doses of DPT and Polio vaccines and one dose of BCG and measles vaccine before the survey |
| ARI treatment |  | The proportion of children under age 5 with suspected pneumonia (cough and difficult breathing NOT due to a problem in the chest and a blocked nose) in the last 2 weeks who received antibiotics |
| Oral rehydration therapy |  | The proportion of children under age 5 with diarrhoea in the last 2 weeks who received oral rehydration therapy (oral rehydration solution, ORS packet, pre-packaged ORS fluid, home-made ORS, any ORS, recommended homemade fluid or increased fluids) and continued feeding during the episode of diarrhoea |

ANC1, at least one antenatal care visit; ANC4, at least four antenatal care visits; IPV, inactivated polio vaccine; OPV, oral polio vaccine; ARI treatment, acute respiratory infection treatment for pneumonia

**Appendix section 3: The definitions of household consumption expenditure and out-of-pocket health payment, estimation of financial burden and incidence of catastrophic health expenditure at different threshold**

**Household consumption expenditure**

On the basis of The World Bank’s guidelines for constructing consumption aggregates [2], a household’s total consumption expenditure was estimated as the aggregate of food consumption expenditure, non-food consumption expenditure, housing expenditure and user cost of durable goods. Non-food consumption expenditure included expenditures for utilities, household goods, clothing, education, transportation, and health. Aggregated expenditure was multiplied with the equivalent household size to estimate total household expenditure.

**OOP health payment**

OOP health payment was set as health expenditure in Household Socio-Economic Survey (HSES) of Iraq in 2006-2007 and 2012. It was the total amount which a household paid when they receive health services. It includes fees for consultation, diagnostic, treatment as well as hospital bills and purchase of medicines including alternative and traditional medicines. In HSES, transportation fees and purchase of special nutritional supplements were not included as proposed by the WHO [3]. It was also multiplied the equivalent household size.

**Measurement of catastrophic health expenditure** [4, 5]

$$Catastrophic health expenditure=\frac{OOP payment}{Household consumption}>X$$

where X is the threshold. The selection of the threshold has varied widely. We understand that there are studies which use 40% of non-food expenditure to capture the fact that poorer households have fewer resources to devote to non-nutritional needs [1]. However, in some countries or areas, other non-discretionary spending, for example, shelter and heating, is relatively even more important than food expenditure, including for poor populations, and any thresholds are not universally applicable [1, 2]. Therefore, in line with Sustainable Development Goal (SDG) UHC indicator 3.8.2 and other studies [4-6], in this study we used 10% of total household consumption expenditure to estimate incidence of catastrophic health expenditure at national-, subnational- and place of residence-levels. In order to compare the findings with other studies, catastrophic health expenditure at the national level was presented for other thresholds using all three denominators in Table A3.

**Table A3: Incidence of catastrophic health expenditure at different threshold**

| **Threshold** | **Catastrophic incidence depending on different threshold** | |
| --- | --- | --- |
|  | **Percent** | **95% Confidence Interval** |
| Total consumption |  |  |
| > 5% | 12.4 | 11.4-13.5 |
| > 10% | 3.3 | 2.8-3.9 |
| Non-food consumption |  |  |
| > 25% | 7.0 | 6.3-7.8 |
| > 40% | 2.2 | 1.8-2.7 |
| Capacity to pay |  |  |
| > 40% | 0.2 | 0.2-0.4 |

**Measurement of impoverishing health payment** [7]

- X is the total expenditure per capita.
- PL is the poverty line, in this study, the region-specific sum of food + non-food component which HSES provided in each survey
- Gross_h_ is the pre-payment poverty head count which is obtained by,

$$Gross_{h}=X <PL$$

- Net_x_ is the per capita total expenditure after paying health care
- OOP is the out-of-pocket payment for health
- Net_h_ is the post payment poverty head count which is obtained by

$$Net_{x}=X-OOP$$

$$Net_{h}=Net_{x}<PL$$

- The poverty head count due to OOP payment is estimated by

$$Diff_{h}=Net_{h}-Gross_{h}$$

**Appendix section 4: Predictor variables for trend and projection analysis and data of internally displaced people and definition of population density**

This study aimed to reflect the associations between displacement and health in assessing UHC trends and projections in Iraq. To identify feasible predictor variables for the study, we considered possible variables such as GDP per capita, and health expenditure per capita, the numbers of internally displaced people (IDPs), returnees, physicians and civilian deaths from violence as well as life expectancy at birth. Through the assessment of data availability, we selected three predictor variables: the numbers of IDPs, population density (both are at the subnational level) and total health expenditure per capita (at the national level) from 2000 to 2018 [8].

**Table A4:** **The list of organisations and data for the numbers of internally displaced people**

| **Organisations** |  | **Data** |
| --- | --- | --- |
| KRSO |  | IDP data 2003-2006 |
| Iraq Ministry of Displacement and Migration |  | IDP data 2008 |
| UNHCR |  | Assessment Report 2000, 2006-2013  Operational Portal Iraq: IDP Situations |
| WFP |  | Comprehensive Food Security and Vulnerability Analysis (CFSVA) 2006, 2008 and 2016 |
| IDMC |  | IDP data 2008 |
| IOM |  | Displacement Tracking Matrix (DTM) Iraq 2014-2019 |
| RI |  | IDP data 2017 |
| OCHA |  | Humanitarian operation dataset 2019 |
| REACH |  | Iraq Intentions Survey Round IV 2019 |

KRSO, Kurdistan Regional Statistics Office; UNHCR, United Nations High Commissioner for Refugees; WFP, World Food Programme; IDMC, Internal Displacement Monitoring Centre; IOM, International Organisation for Migration; RI, Refugees International; OCHA, The Office for the Coordination of Humanitarian Affairs

**Population density**

Population density of each governorate was the sum of the numbers of residents and IDPs in each governorate, divided by the size of the area.

$$Population density=\frac{number of residence+number of IDPs}{size of area}$$

If the numbers of IDPs and numbers of residents, including the official estimates, were not available, they were estimated out of the numbers in the previous/following years. When inconsistent numbers were found between different organisations, most probable or modest data were used. In this study, returnees were included in the residence due to limited data, despite the fact that there were returnees who stayed not at home but in the IDP camps in their home governorates.

**Appendix section 5. Predictor variables used in the determinant analyses**

**Table A5.1: Predictor variables used in the analysis of the determinants of health service coverage**

| **Variables** |  | **Definition** |
| --- | --- | --- |
| Age of woman, years |  | Continuous in number |
| Education of woman |  | 1=No education, 2=Primary, 3=Secondary, 4=Higher, 5=Others |
| Birth order |  | 1=No birth before, 2=Once, 3=Two or three times, 4=Four and over |
| Antenatal care, times ^a^ |  | Continuous in number |
| Gender of the last newborn ^a^ |  | 1=Male, 2=Female |
| Wealth quintile of household |  | 1=Q1 (poorest), 2=Q2, 3=Q3, 4=Q4, 5=Q5 (richest) |
| Place of residence |  | 1=Urban, 2= Rural |

Note: ^a^ It was applied for full immunisation, acute respiratory infection treatment for pneumonia and oral rehydration therapy. For a proxy indicator to measure relative wealth, we used wealth index which MICS provided in their individual dataset.

**Table A5.2: Predictor variables used in the analysis of the determinants of catastrophic expenditure and impoverishment**

| **Variables** |  | **Definition** |
| --- | --- | --- |
| Age of household head, years |  | Continuous in number |
| Gender of household head |  | 1=Male, 2=Female |
| Education of household head |  | 1=No education, 2=Primary, 3=Secondary, 4=Higher, 5=Others |
| Number of household members aged under 5 years old |  | Number |
| Number of household members aged over 65 years old |  | Number |
| Wealth quintile of household |  | 1=Q1 (poorest), 2=Q2, 3=Q3, 4=Q4, 5=Q5 (richest) |
| Place of residence |  | 1=Urban, 2= Rural |
| Survey year |  | 1=2007, 2=2012 |

Note: The details of construction of household wealth quintile are described below.

**Household economic status for financial risk protection indicator analysis**

We followed other studies and constructed a household wealth index based on the information of household consumption expenditure for HSES in Iraq (2007, 2012) [2, 3]. Total consumption expenditure of the household was used as effective income since it is a more accurate reflection of purchasing power than income reported in household surveys [9, 10]. For each household, an equivalised per capita expenditure was estimated by dividing the total expenditure consumption by the equivalent household size [3]. Households were ranked into quintiles based on the equivalised per capita expenditure. The lowest 20% was regarded as the poorest quintile (Q1) and the highest 20% as the richest (Q5) [2, 11].

**Appendix section 6. Statistical analysis**

**Bayesian approach**

Bayesian approach addresses the issues of limited data points across all provenances and is used and favoured when we aim to project probabilities. This unique advantage of Bayesian approach to produce probabilistic-oriented inferences was key in conducting our study. Bayesian approach has been increasingly applied especially to ecological studies. Ecological modelling is characterised by high uncertainty because of complex and often unknown cause-effect relationships among variables. Therefore, a probabilistic approach is needed to yield distributions of possible outcomes. Bayesian method also has an ability to combine prior knowledge about parameters with evidence from data and is favoured for analysis of hierarchical models [12]. It enables flexibility in specifying hierarchical structures of parameters using priors; ability to manage small samples and model misspecification; explicit handling of uncertainty; and intuitive interpretations of results (credible interval versus confidence interval) [13].

Predictor variables used in the trend analysis were projected up to 2030 using a Bayesian hierarchical regression model:

$$y_{ijk}= \alpha_{jk}+ \beta_{j}{year}_{i}+ \varepsilon_{ijk}$$

where y is the logit-transformed probability of the population density, population movement, or total per-capita health expenditure variables for *i:*th year, *j*:th governorate and *k*:th region.$\alpha_{jk}$ is the random component of the *j:*th governorate in *k:*th region. $\beta_{j}$ is the random component of the time slopes (year) for *j:*th governorate. $\varepsilon_{ijk}$ is the residual of the hierarchical model. The projection estimates were merged with health service indicators to develop the complete data set.

Considering the hierarchical structure of the data, the Bayesian hierarchical linear regression model was developed with random intercepts and random slopes at governorate level. All individual observations were nested in their respective governorate and all governorates were again nested as a country. Our projection model was based on the assumption of the unchanged policy in the near future. The following model was used to estimate the trend in, and projection of, health service indicators up to 2030 at governorate level:

$$y_{ijk}= \alpha_{jk}+ \beta_{j}{year}_{i}+ \delta_{j1}{PD}_{ij}+ \delta_{j2}{PM}_{ij}+ \gamma_{j}{THE}_{i}+ \varepsilon_{ijk}$$

where y is the logit-transformed probability of health service indicator in *i*:th year for *j*:th governorate in *k*:th region. $\alpha_{jk}$ is the random component of the *j:*th governorate in *k:*th region. $\beta_{j}$ is the random component of the time slopes (year) for *j*:th governorate. $\delta_{j1}$ is the random coefficient of the population density (PD) in *j*:th governorate. $\delta_{j2}$ is the random coefficient of the population movement (PM) in *j*:th governorate. $\gamma_{j}$ is the random coefficient of total per-capita health expenditure (THE) for *j*:th governorate. $\varepsilon_{ijk}$ is the residual of the hierarchical model. The other health service indicators were developed separately by using the urban-rural and wealth-quintile specific health service indicators. In the models, the same covariates included in the previous model were applied.

The governorate-level mean, residence-level mean, and quintile-level mean were assumed to be normally distributed and non-informative prior was applied. The model assumed that the effects of predictors were the same across governorates. The predictor variables were determined based on the previous literature, correlation, and Deviance Information Criteria (DIC).

Trace plots were checked visually to assess convergence of Markov chain Monte Carlo (MCMC) output for each of the Bayesian models. When the outputs from two chains adjoined, the posterior samples were considered to have converged [14]. A potential scale reduction factor (PSRF) is used in the Gelman-Rubin diagnostic, where a PSRF value close to 1 indicated convergence, and a PSRF value less than 1.02 identified convergence failure [14]. To examine the validity of the models, we plotted our predictions versus the observed data across governorates and by year. We calculated bias (mean error), total variance (root-mean-square error), and 95% data coverage within prediction intervals.

**Wealth-based inequality**

Wealth-based inequalities in health service coverage and incidence of catastrophic health expenditure were performed using slope index of inequality (SII) and relative index of inequality (RII). SII measures the absolute difference in intervention coverage or catastrophic health expenditure between the richest households and the poorest households. A weighted sample of the entire population was ranked from the poorest quintile to the richest quintile. The outcomes of health service and financial risk protection indicators were regressed against the midpoint value for quintile groups by using regression model. The difference between the estimated values at the richest households (𝑣1) and the poorest households (𝑣0) generates the SII value: 𝑆𝐼𝐼 = 𝑣1 − 𝑣0 [15]. A positive SII value indicates that rich households have either higher intervention coverage or higher financial catastrophe than poor households. RII is a weighted measure of inequality which represents the ratio of estimated values of a health indicator of the richest households to the poorest households. The ratio of the estimated values at the richest households (𝑣1) to the poorest households (𝑣0) generates the RII value: 𝑅𝐼𝐼 = 𝑣1/𝑣0 [15]. When there is no inequality, RII takes the value 1. An RII value greater than 1 indicates pro-rich inequality and a value smaller than 1 indicates pro-poor inequality.

**Appendix section 7. National-level health service coverage by wealth quintile and slope index of inequality**

**Table A7: Slope index of inequality of health service coverage at the national level in Iraq, 2000-2030**

| **Health service indicators** | **SII (95% CI)** | | | |
| --- | --- | --- | --- | --- |
|  | **2000** | **2018** | **2030** |  |
| ANC1 | 30.7 (11.6-49.8) | 20.2 (14.9-25.4) | 17.6 (12.5-22.7) |  |
| ANC4 | 28.9 (9.3-48.6) | 29.9 (22.2-37.7) | 15.4 (9.4-21.3) |  |
| Institutional delivery | 26.5 (4.8-48.2) | 8.8 (5.5-12.1) | 2.8 (1.6-4.0) |  |
| Skilled birth attendance | 38.2 (21.2-55.3) | 6.9 (3.4-10.3) | 1.8 (0.6-2.9) |  |
| Full immunisation | 30.6 (12.1-12.1) | 38.9 (26.2-51.6) | 20.0 (20.0-20.0) |  |
| BCG | 11.1 (3.7-18.4) | 7.6 (3.6-11.5) | 8.9 (8.9-12.2) |  |
| DTP3 | 29.8 (13.3-46.3) | 32.9 (22.7-43.0) | 21.4 (16.7-26.1) |  |
| Polio3 | 29.4 (11.9-46.9) | 28.0 (16.0-39.9) | 12.7 (7.8-17.5) |  |
| Measles | 25.6 (11.2-39.9) | 28.2 (14.7-41.8) | 18.6 (14.3-23.0) |  |
| ARI treatment | 0.4 (-0.6-1.4) | 36.0 (16.9-55.0) | 7.7 (3.3-12.1) |  |
| Oral rehydration therapy | -2.1 (-3.7-0.4) | 36.2 (12.7-59.7) | 9.6 (4.4-14.7) |  |
| Improved water sources | 66.5 (56.1-76.8) | 2.7 (0.2-5.3) | 4.9 (2.4-7.3) |  |
| Adequate sanitation | 55.5 (37.9-73.1) | 6.9 (1.1-12.6) | 0.0 (0.0-0.0) |  |

SII, slope index of inequality; CI, confidence interval; FPNS, family planning needs satisfied; ANC1, at least one antenatal care visit; ANC4, at least four antenatal care visits; ARI treatment, acute respiratory infection treatment for pneumonia

**Appendix section 8. Subnational-level catastrophic health expenditure by place of residence**


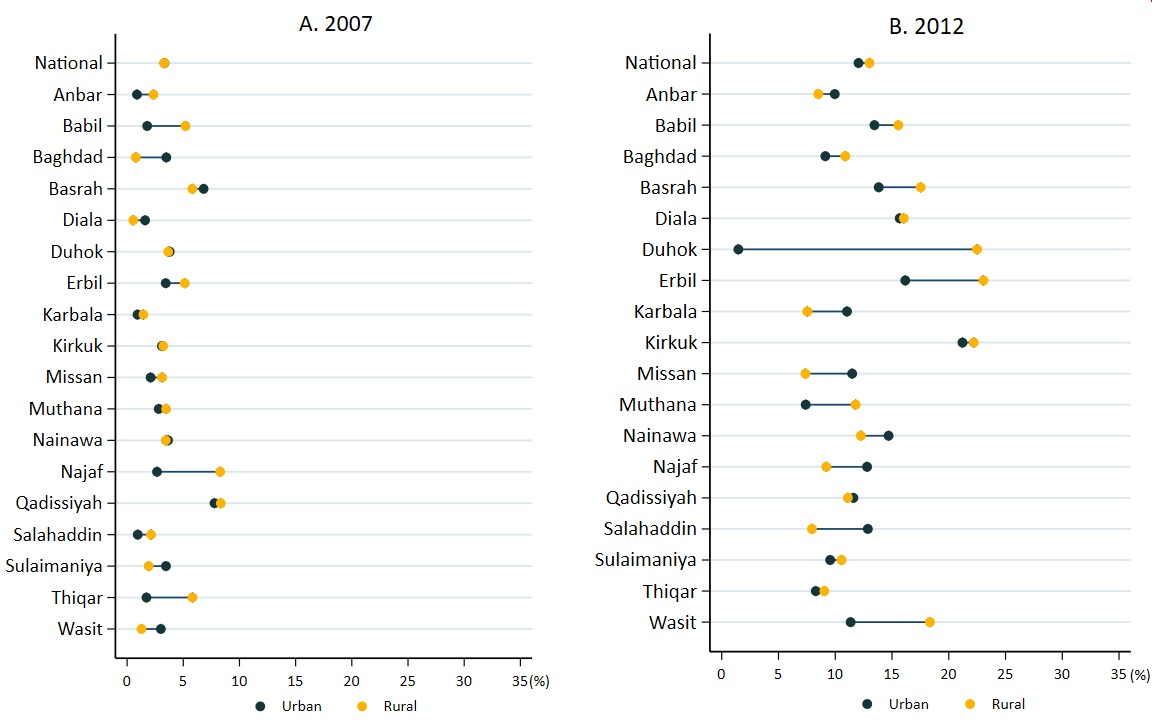


**Figure A8: Residence-specific incidence of catastrophic health expenditure at the national and subnational level in Iraq, 2007 and 2012**

**Appendix section 9. Subnational-level catastrophic health expenditure: slope index of inequality and wealth quintile-specific incidence**


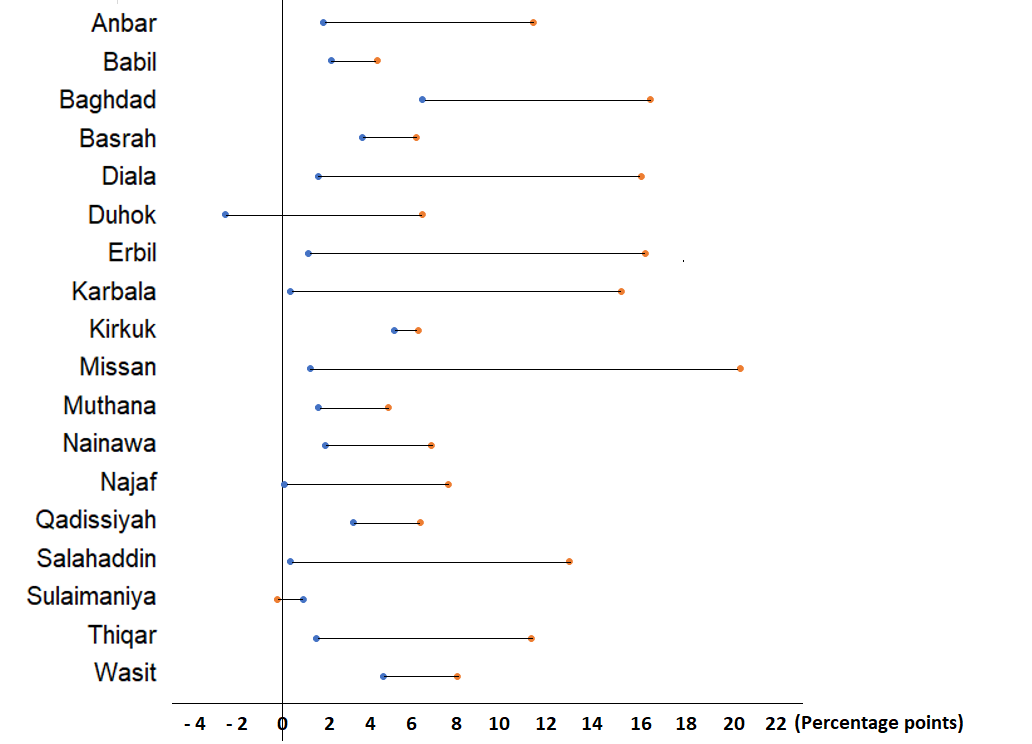


● **2007** 　　● **2012**

**Figure A9.1: Slope index of inequality in the subnational-level catastrophic health expenditure in Iraq, 2007 and 2012**


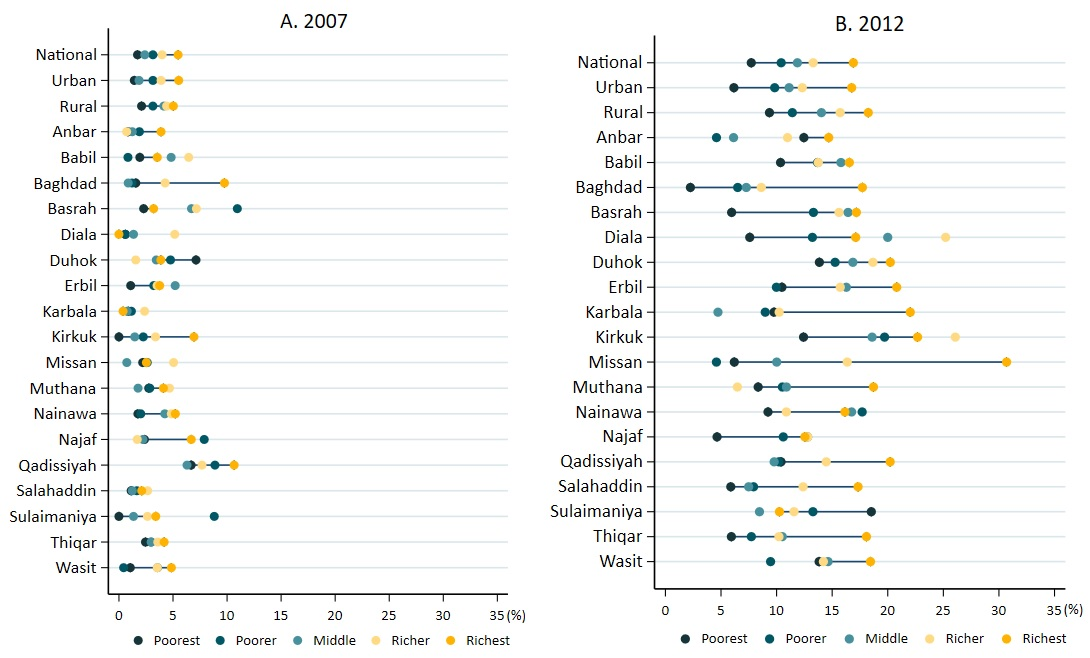


**Figure A9.2: Wealth quintile-specific incidence of catastrophic health expenditure at the national and subnational level in Iraq, 2007 and 2012**

**References**

1. Central Organization for Statistics & Information Technology and Kurdistan Regional Statistics Office: **Multiple Indicator Cluster Survey Iraq 2018.** 2019.

2. O'Donnell O, van Doorslaer E, Wagstaff A, Lindelow M: *Analyzing Health Equity Using Household Survey Data: A Guide to Techniques and Their Implementation.* Washington, DC: The World Bank; 2008.

3. World Health Organization: **Distribution of health payments and catastrophic expenditures Methodology / by Ke Xu.** Geneva: World Health Organization; 2005.

4. World Health Organisation: **Primary Health Care on the Road to Universal Health Coverage 2019 MONITORING REPORT.** Conference edition edition. pp. 162. Geneva: World Health Organisation; 2019:162.

5. Wagstaff A, Neelsen S: **A comprehensive assessment of universal health coverage in 111 countries: a retrospective observational study.** *The Lancet Global Health* 2020, **8:**e39-e49.

6. Wagstaff A, Flores G, Hsu J, Smitz M-F, Chepynoga K, Buisman LR, van Wilgenburg K, Eozenou P: **Progress on catastrophic health spending in 133 countries: a retrospective observational study.** *The Lancet Global Health* 2018, **6:**e169-e179.

7. Swe KT, Rahman MM, Rahman MS, Saito E, Abe SK, Gilmour S, Shibuya K: **Cost and economic burden of illness over 15 years in Nepal: A comparative analysis.** *PLoS One* 2018, **13:**e0194564.

8. **Global Health Expenditure Database** [<https://apps.who.int/nha/database/ViewData/Indicators/en>]

9. Xu K, Klavus J, Kawabata K, Evans D, Hanvoravongchai P, Ortiz J, Zeramdini R, Murray C: *Household Health System Contributions and Capacity to Pay: Definitional, Empirical, and Technical Challenges.* Geneva: World Health Organisation; 2003.

10. Xu K, Evans DB, Kawabata K, Zeramdini R, Klavus J, Murray CJ: **Household catastrophic health expenditure: a multicountry analysis.** *Lancet* 2003, **362:**111-117.

11. Deaton AZ, Salman; Deaton, Angus Zaidi, Salman,: **Guidelines for constructing consumption aggregates for welfare analysis (English).** In *Living standards measurement study (LSMS) working paper*. Washington, D.C: The World Bank; 2002.

12. Arhonditsis GB, Stow CA, Steinberg LJ, Kenney MA, Lathrop RC, McBride SJ, Reckhow KH: **Exploring ecological patterns with structural equation modeling and Bayesian analysis.** *Ecological Modelling* 2006, **192:**385-409.

13. Grzenda W: **The Advantages of Bayesian Methods over Classical Methods in the Context of Credible Intervals.** *Information Systems in Management* 2015, **4:**11.

14. Gelman A CJ, Stern HS, Rubin DB.: *Bayesian Data Analysis.* Third Edition edn. USA: Chapman and Hall/CRC; 2013.

15. World Health Organisation: **Health Equity Assessment Toolkit (HEAT): Software for exploring and comparing health inequalities in countries.** Built-in Database Edition edition. Geneva; 2017.
